# Fundamental and Applicative Aspects of the Unfolded Protein Response in Yeasts

**DOI:** 10.3390/jof9100989

**Published:** 2023-10-05

**Authors:** Yuki Ishiwata-Kimata, Yukio Kimata

**Affiliations:** Division of Biological Science, Graduate School of Science and Technology, Nara Institute of Science and Technology, Nara 630-0192, Japan

**Keywords:** yeast, endoplasmic reticulum, stress response, unfolded protein response

## Abstract

Upon the dysfunction or functional shortage of the endoplasmic reticulum (ER), namely, ER stress, eukaryotic cells commonly provoke a protective gene expression program called the unfolded protein response (UPR). The molecular mechanism of UPR has been uncovered through frontier genetic studies using *Saccharomyces cerevisiae* as a model organism. Ire1 is an ER-located transmembrane protein that directly senses ER stress and is activated as an RNase. During ER stress, Ire1 promotes the splicing of *HAC1* mRNA, which is then translated into a transcription factor that induces the expression of various genes, including those encoding ER-located molecular chaperones and protein modification enzymes. While this mainstream intracellular UPR signaling pathway was elucidated in the 1990s, new intriguing insights have been gained up to now. For instance, various additional factors allow UPR evocation strictly in response to ER stress. The UPR machineries in other yeasts and fungi, including pathogenic species, are another important research topic. Moreover, industrially beneficial yeast strains carrying an enforced and enlarged ER have been produced through the artificial and constitutive induction of the UPR. In this article, we review canonical and up-to-date insights concerning the yeast UPR, mainly from the viewpoint of the functions and regulation of Ire1 and *HAC1*.

## 1. Introduction

The size, shape, and function of eukaryotic organelles can vary depending on the cell type and environmental cues. For example, mammalian brown adipocytes are rich in mitochondria for thermogenesis [1]. In the methylotrophic yeast *Pichia pastoris* (syn. *Komagataella* spp.), peroxisomes proliferate when methanol is used as the carbon source [2]. Eukaryotic cells carry various intracellular signaling pathways that ultimately enlarge and/or enforce their target organelles. The unfolded protein response (UPR) is induced by dysfunction or the functional shortage of the endoplasmic reticulum (ER), namely, ER stress.

The yeast *Saccharomyces cerevisiae* is one of the most prominent model organisms in the field of cell biology. It also has a long history as an ethanol-fermenting microorganism in the field of food engineering and, more recently, in biofuel production. Moreover, other yeast and fungal species have unique characteristics that allow their use for various industrial purposes. For instance, *P. pastoris* is frequently used as a host for heterologous protein production, partly because it has an extremely robust and inducible gene expression promoter [3,4]. In clinical science, the pathogenicity of some yeast and fungal species is an important problem. In this review article, we present historical and up-to-date insights into the yeast UPR from the viewpoints of both fundamental and applied sciences.

## 2. The Endoplasmic Reticulum (ER)

Eukaryotic cells commonly carry the ER, which is surrounded by a lipid bilayer membrane. In many cell types, the ER exists as flat sheets or a tubular network and largely expands in the cytoplasm. As a general understanding, the ER is composed of two regions: a rough ER and a smooth ER. The rough ER is called so because many ribosomes are attached to the cytosolic side of the ER membrane.

**Figure 1:**
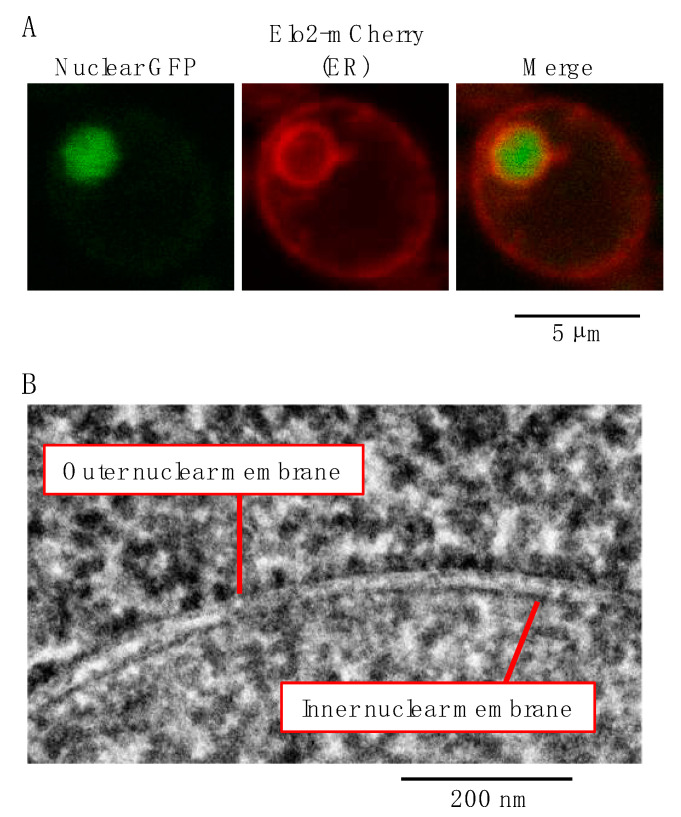
The ER in *S. cerevisiae* cells. (**A**) *S. cerevisiae* FY8 cells expressing both a nuclear-localized version of green fluorescent protein (GFP) and Elo2 (an ER-located transmembrane protein) fused to mCherry were observed under a confocal fluorescence microscope. (**B**) A lead-stained ultrathin section of an *S. cerevisiae* BY4742 cell was observed under a transmission electron microscope. Ribosomes attached to the outer nuclear membrane.

The ER morphology of *S. cerevisiae* is likely to be simpler than that of higher eukaryotes [5]. Via the fluorescence microscopy of *S. cerevisiae* cells producing an ER-marker fluorescent protein, the ER appears to be a “double ring” (Figure 1A). The inner ring corresponds to the nuclear ER, which is identical to the nuclear envelope that covers nuclear genomic DNA. The outer ring is called the cortical ER, which is juxtaposed with the plasma membrane. Unlike that of higher eukaryotes, the ER of *S. cerevisiae* is not clearly partitioned into rough and smooth ERs. Under electron microscopy, ribosomes appeared to be uniformly attached to the cytosolic surface of the cortical ER and the outer nuclear membrane (Figure 1B).

A well-known role of ER is to serve as a site where secretory and transmembrane proteins are folded and assembled. Ribosomes that translate mRNAs encoding ER client proteins are attached to the ER membrane and plunge nascent peptides into the ER. After being correctly folded and assembled, ER client proteins are packed into transport vesicles for their transportation to the Golgi apparatus, where they are sorted for further transportation to the cell surface or other organelles [6].

To support protein folding, the ER contains various molecular chaperones, including BiP. While BiP was initially discovered as a protein that binds to premature immunoglobulin proteins in the mammalian ER, *S. cerevisiae* cells also carry it [7,8], implying it has a ubiquitous role. BiP is an HSP70-family molecular chaperone and is known to play multiple roles [9]. *S. cerevisiae* BiP is named kar2. Kar2 is reported to act as a molecular ratchet for nascent peptides during their translocation across the ER membrane [10]. Moreover, Kar2, as well as other molecular chaperones, monitors, assists, and controls protein folding [11]. Kar2 is associated with unfolded and/or unassembled ER client proteins, which are frequently returned to the cytosol and degraded by the proteasome (ER-associated degradation, ERAD) [12].

Protein folding in the ER is frequently accompanied by intra- or intermolecular disulfide bond formation between cysteine residues, namely, oxidative protein folding, which is accomplished by some ER-located enzymes, including Pdi1 and Ero1 [13]. Moreover, many ER client proteins are glycosylated in the ER.

In addition, the ER membrane is the site in which lipidic molecules are biosynthesized. Phospholipids are mainly metabolized on the ER membrane, resulting in the expansion of the ER membrane, and transported to other organelles and the cell surface via vesicle transport. Moreover, lipid droplets, in which neutral lipids are stored, emerge from the ER [14]. Calcium ion storage is believed to be another important role of h=the ER. However, the calcium ion concentration in the *S. cerevisiae* ER is fairly low, which suggests that it does not store high amounts of calcium ions [15].

## 3. UPR Inducing and Repressing Mechanisms in *S. cerevisiae* Cells

Kozutsumi et al. [16] reported that in mammalian cells, BiP and another ER-located molecular chaperone, GRP94, are transcriptionally induced alongside the accumulation of misfolded or unfolded proteins in the ER. Subsequently, this cellular response, currently known as the unfolded protein response (UPR) or ER stress response, was further explored using *S. cerevisiae* as a model organism. Two key factors in the intracellular UPR signaling pathway, Ire1 and *HAC1*, have been discovered through yeast genetic studies [17,18,19].

**Figure 2:**
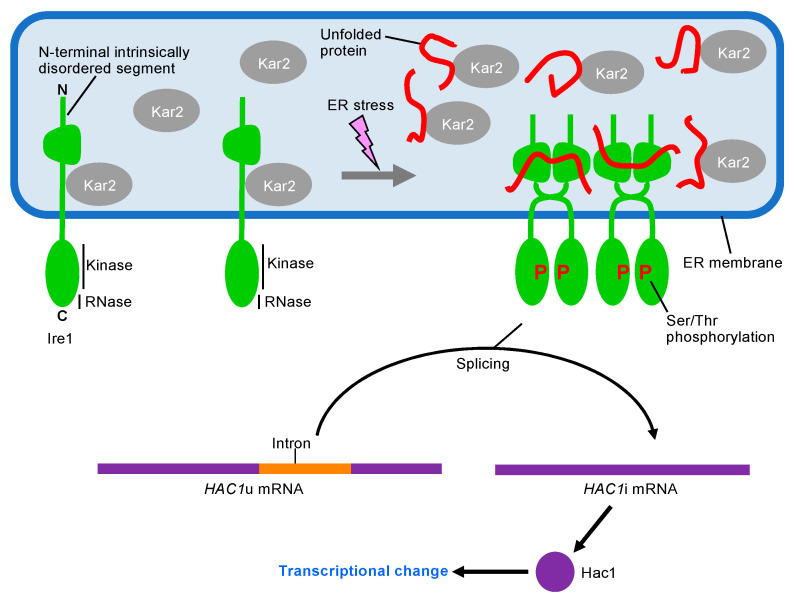
Ire1- and *HAC1*-dependent UPR in *S. cerevisiae* cells. Kar2 binds to Ire1, which then remains non-self-associated, under non-stress conditions. The N-terminal intrinsically disordered segment of Ire1 also inhibits its self-association. ER stress causes the dissociation of Kar2 from Ire1, which is then self-associated. Moreover, Ire1 dimers capture ER-accumulated unfolded proteins and form high-order oligomers. The self-association of Ire1 promotes its auto-phosphorylation. Oligomerized and auto-phosphorylated Ire1 exhibits strong RNase activity. In addition, ADP is captured by the kinase domain of Ire1 as an activation ligand (not shown in this figure). The *HAC1* mRNA is spliced depending on the RNase activity of Ire1, and then translated into a transcription factor protein that is responsible for the transcriptional change in UPR.

Ire1 is an ER-located type-I transmembrane endoribonuclease that acts as an ER stress sensor [20]. As shown in Figure 2, while *HAC1* mRNA is transcribed as a precursor form containing an intron sequence (*HAC1*u; “u” means “uninduced”), it undergoes an Ire1-dependent splicing reaction and is converted to the mature form (*HAC1*i; “i” means “induced”) under ER stress conditions [19]. *HAC1*i mRNA is then translated into the bZIP transcription factor Hac1, which is responsible for the UPR.

In addition to Ire1 and *HAC1*, some other factors are essential for or modulate the UPR signaling pathway in *S. cerevisiae* cells. For instance, the tRNA ligase Trl1 (syn. Rlg1) is involved in the ligation of two exon fragments of *HAC1* mRNA in the Ire1-dependent mRNA-splicing reaction [21]. Moreover, Ghosh et al. [22] reported that Pal1 and Pal2, which are known to be involved in endocytosis, bind to the 3’-untranslated region (UTR) of *HAC1* mRNA and promote its splicing when they are phosphorylated by the protein kinases Kin1 and Kin2.

How is Ire1 activated in response to ER stress? We and others have indicated that the luminal domain of Ire1 directly detects unfolded proteins accumulated in the ER, leading to the high-order oligomerization and punctate distribution of Ire1 (Figure 2) [23,24,25]. X-ray crystallographic and biochemical analyses of the cytosolic domain of Ire1 demonstrated that it exerts strong endoribonuclease activity when it is highly self-oligomerized [26]. Moreover, *HAC1*u mRNA is likely to be actively recruited to the Ire1 puncta, where it is converted into *HAC1*i mRNA [27,28].

Meanwhile, Ire1 additionally undergoes multiple regulatory events, resulting in UPR induction being strictly dependent on ER stress. Under non-stress conditions, BiP or Kar2 is associated with the luminal domain of Ire1 to inhibit its self-association and activation [29,30]. Although BiP or Kar2 dissociates from Ire1 upon ER stress (Figure 2), this event alone is unlikely to be sufficient to activate Ire1. Ire1 mutants not carrying the Kar2-binding site are upregulated by ER stress, similar to wild-type Ire1 in *S. cerevisiae* cells [31]. Furthermore, *S. cerevisiae* Ire1 carries an N-terminal intrinsically disordered segment, which inhibits the self-association of Ire1 in a Kar2-independent manner under non-stress conditions [32].

In addition to the endoribonuclease motif, the cytosolic domain of Ire1 contains a Ser/Thr protein kinase motif, which is responsible for the autophosphorylation of Ire1 (Figure 2) [33]. As mentioned above, Ire1 self-associates during ER stress, leading to its trans-phosphorylation (one Ire1 molecule autophosphorylates another Ire1 molecule) in an ER-stress-dependent manner [33,34]. The autophosphorylation of Ire1 changes its structure to exert strong endoribonuclease activity [35]. Moreover, ADP binds to the kinase motif of Ire1 as an activation ligand [26,35,36]. Consequently, Ire1 exhibits strong activity in splicing *HAC1*u mRNA and inducing UPR in unhealthy cells carrying high levels of ADP [37].

Some mechanisms exist that subside the UPR after peak induction. According to Chawla et al. [38] and Rubio et al. [39], UPR attenuation involves the dephosphorylation of Ire1. Furthermore, as a negative feedback mechanism, BiP induced by the UPR re-associates with and downregulates Ire1 [40]. More recently, Matabishi-Bibi et al. [41] reported that Isw1 retains *HAC1* mRNAs in the nucleus to subside the UPR.

*HAC1*u mRNA is likely to be translated into a weak-functional transcription factor because its artificial overexpression partially rescued the UPR-deficient phenotype of the *IRE1*-knockout strain [19]. However, *HAC1*u mRNA is poorly translated because of its intramolecular hybridization between the 5′-UTR sequence and the intron sequence [42,43]. Nevertheless, this translation repression is incomplete, and the leaky translation product of *HAC1*u mRNA is rapidly digested by the proteasome [44,45]. According to Sarkar et al. [46], *HAC1*u mRNA is rapidly digested under non-stress conditions. In addition, Uppala et al. [47] proposed a role of certain signaling pathways that repress *HAC1* mRNA translation independent of its splicing.

Taken together, the UPR level is tightly controlled to avoid an inappropriate UPR under no or weak stress conditions. The inappropriate activation of Ire1 harms *S. cerevisiae* cells [37,38,39].

## 4. Scenes in which the UPR Is Provoked in *S. cerevisiae* Cells

As its name denotes, the UPR has been believed to be a cellular response to cope with the accumulation of unfolded proteins in the ER. *S. cerevisiae* cells provoke a potent UPR in the presence of the thiol-reducing agent dithiothreitol (DTT) or the N-glycosylation-inhibiting antibiotic tunicamycin, probably because these chemicals cause the ER accumulation of aberrant proteins. Strikingly, UPR induction by DTT or tunicamycin is compromised when Ire1 carries a luminal domain mutation that impairs its ability to sense unfolded proteins accumulated in the ER [23,48].

**Figure 3:**
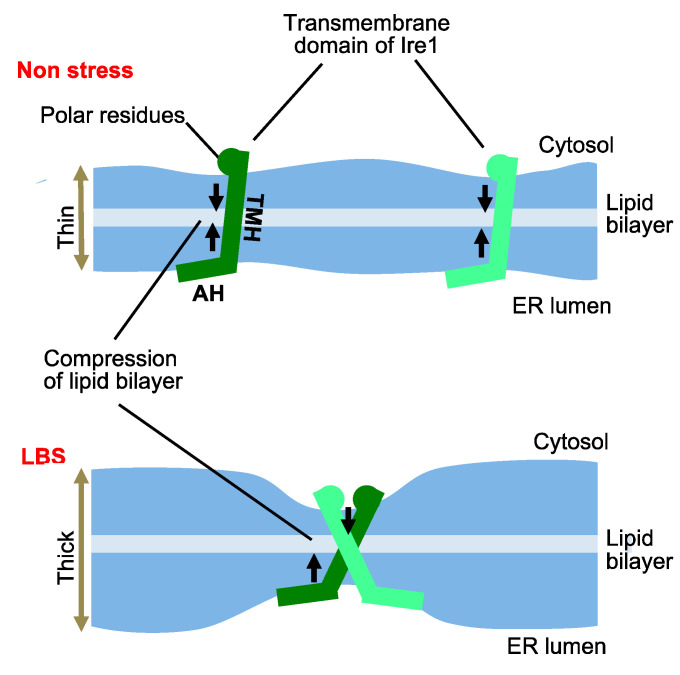
Self-association of Ire1 during LBS. The transmembrane helix (TMH) of Ire1 is interposed between an amphipathic helix (AH) and polar residues. Halbleib et al. [51] proposed that this causes the compression of the lipid bilayer. LBS increases the thickness and unevenness of the lipid bilayer, leading to the assembly of Ire1, which decreases the free-energy cost of lipid bilayer compression.

On the other hand, the depletion of inositol from culture media also considerably induces the UPR [49]. Intriguingly, even in the absence of the luminal domain, Ire1 is upregulated by inositol depletion and genetic mutations that are likely to affect the phospholipid composition of biological membranes [48,50]. Therefore, such stress stimuli, namely, lipid bilayer stress (LBS) and the ER accumulation of unfolded proteins, are likely to be detected by Ire1 and induce an UPR in different manners. According to Halbleib et al. [51], the transmembrane domain of Ire1 takes a unique form that is responsible for the self-association of Ire1 in response to LBS (Figure 3).

The basic architecture of Ire1 dimers does not seem to differ regardless of the primary cause of stress [52]. Nevertheless, Ire1 has been reported to be activated not as high-order oligomers, but as dimers in response to LBS [50]. Dimeric Ire1 is unlikely to exert RNase activity as robustly as oligomeric Ire1 does [26,53]. These insights explain a molecular basis by which an overly strong UPR is avoided during LBS.

A prominent cause that triggers the UPR via the induction of LBS is the saturation of membrane lipids, which considerably affects membrane thickness and fluidity [54]. Moreover, Micoogullari et al. [55] proposed that the UPR is provoked by defects in very long-chain fatty acid (VLCFA) metabolism. It is also likely that shortage of phosphatidylcholine (PC), a major membrane-constituting phospholipid, induces the UPR [56]. In *S. cerevisiae* cells, impairments in VLCFA metabolism and PC biosynthesis result in fatty acid saturation, which can be a common cause of this [55,57]. PC is partly biosynthesized via the sequential methylation of phosphatidylethanolamine. Ishiwata-Kimata et al. [58] demonstrated that an intermediate product of this reaction, phosphatidyl-*N*-monomethylethanolamine, per se induces ER stress and provokes the UPR.

Tran et al. [59] reported that the ER accumulation of unfolded proteins and LBS, both of which induce the UPR, are distinguishable using Ire1 mutants. As described above, Ire1 is impaired in the detection of unfolded proteins that accumulate in the ER when carrying a luminal domain mutation [23,48]. On the other hand, transmembrane-domain mutations of Ire1 compromise its ability to be activated by LBS [50,51].

ER-client soluble proteins cannot exit the ER when carrying mutations that impair their proper folding. We and others have proposed that such a protein induces the UPR through its direct association with the luminal domain of Ire1 [24,48]. According to Phuong et al. [60], a mutant form of a transmembrane protein triggers the UPR via the induction of LBS rather than via its direct association with Ire1.

In addition to these pure laboratory conditions, the UPR is triggered under pseudo-natural or industrial situations. For instance, *S. cerevisiae* produces ethanol, which is harmful and induces the UPR in *S. cerevisiae* [59,61,62]. Navarro-Tapia et al. [62] proposed that ethanol affects membrane lipid properties, leading to the UPR induction. Moreover, it is also possible that ethanol impairs protein folding in the ER [59,61]. Another intriguing characteristic of *S. cerevisiae* is that it initiates aerobic respiration upon the deprivation of fermentable sugars. Tran et al. [63] reported that under this physiological change, namely a diauxic shift, the UPR is induced in *S. cerevisiae* cells. Cadmium, a prominent environmental pollutant, is known to provoke the UPR in many organisms. Le et al. [64] demonstrated that in *S. cerevisiae* cells, cadmium ions impair protein folding in the ER, leading to the activation of Ire1.

As mentioned above, a reductive environment that inhibits disulfide-bond formation causes strong ER stress. Conversely, it may be unlikely that oxidative stress alone induces ER stress and the UPR [63]. Meanwhile, UPR induction upon diauxic shift involves reactive oxygen species [63].

When the UPR signaling pathway is halted by *IRE1* or *HAC1* knockout mutations, *S. cerevisiae* cells exhibit hypersensitivity to the aforementioned ER stress stimuli. Moreover, the UPR deficiency impairs mitochondrial expansion upon diauxic shift and reduces the chronological lifespan [63,65].

## 5. UPR Target Genes in *S. cerevisiae* Cells

Whereas the Ire1-*HAC1* signaling pathway of the UPR was initially found to induce ER-located molecular chaperones and protein-folding enzymes in *S. cerevisiae* cells, genome-wide transcriptome analyses have indicated that a wider variety of target genes are controlled by the UPR. Using a DNA microarray technique, Travers et al. [66] investigated the gene expression profile under ER stress conditions and its alteration by *IRE1* or *HAC1* deletion mutations. Moreover, Kimata et al. [67] monitored the gene expression profile in cells constitutively expressing the active form of *HAC1* mRNA, namely, *HAC1*i mRNA.

**Figure 4:**
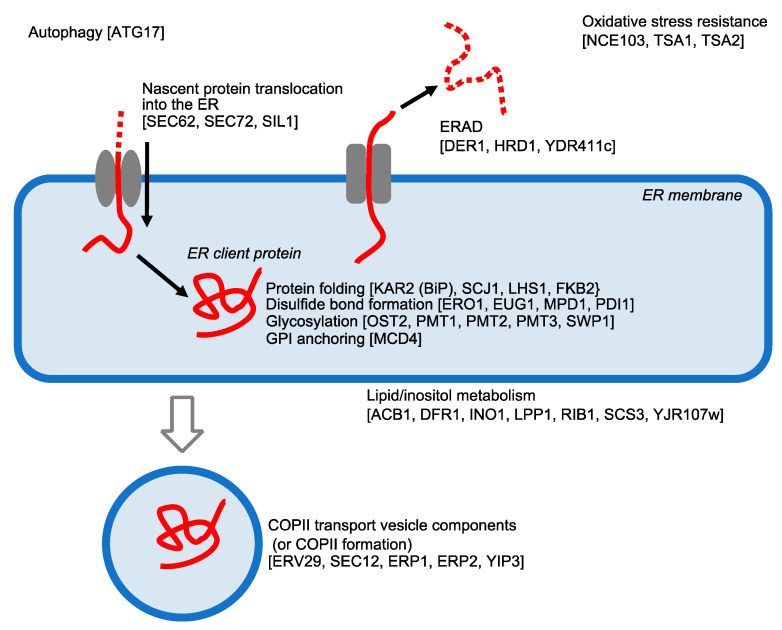
Genes induced by the UPR in *S. cerevisiae* cells. Based on Kimata et al. [67], the genes induced by the Ire1- and *HAC1*-dependent UPR are exemplified.

Figure 4 shows that the genes induced by the UPR predominantly encode a wide variety of proteins that function in the ER and/or protein secretory pathway. In other words, the functions of the ER are totally enforced by the UPR, which is considered as a cellular response to cope with ER stress. The UPR has a large number of target genes possibly and partly because the Hac1 protein, which is the translation product of *HAC1*i mRNA, recognizes two distinct gene-promoter motifs [68].

In agreement with the insight that the UPR induces some genes involved in ERAD (Figure 4), efficient ERAD requires an intact UPR [66,69]. Moreover, UPR results in an expansion of the ER size, which contributes to the alleviation of ER stress [70]. On the other hand, it is also likely that the UPR induces a type of autophagy that selectively digests the ER, namely ER-phagy, possibly to remove damaged parts of the ER [71]. Another noteworthy upregulating target of the UPR pathway is *HAC1* gene, implying a positive feedback regulatory loop [72].

**Figure 5:**
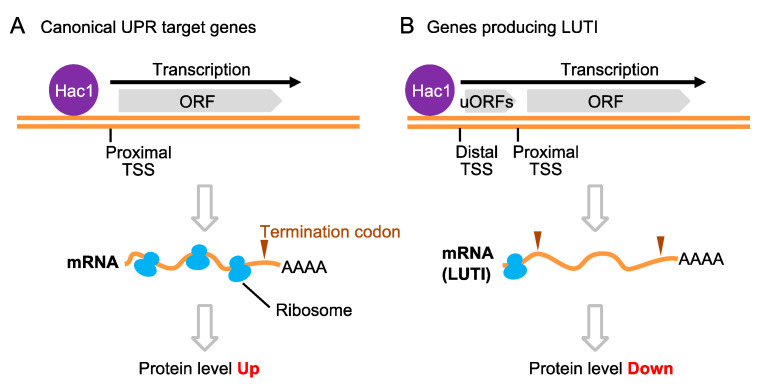
Repression of gene expression through forming long, un-decoded transcript isoforms (LUTIs) by Hac1. (**A**) For the canonical UPR, Hac1 induces the transcription of various target genes from the transcription start site (TSS) proximal to the open reading frame (ORF). This upregulates protein production by Hac1-target genes. (**B**) In the case of some other genes, Hac1 causes transcription from the unconventional TSS distal to the OFR. This yields LUTIs, on which ribosomes scan only the upstream ORFs (uORFs), and results in the downregulation of protein production from Hac1-target genes.

Van Dalfsen et al. [73] and Matsuki et al. [74] proposed that Hac1 binds to the distal transcription initiation sites of some genes, leading to the production of long, un-decoded transcripts (Figure 5). This phenomenon results in translational repression of target genes and, according to Van Dalfsen et al. [73], contributes to the reduction of cellular respiration under ER stress conditions.

At least in the case of *S. cerevisiae*, the functions of Ire1 and *HAC1* are very interdependent. As described above, *HAC1u* mRNA activity is severely repressed. In addition, genome-wide analyses performed by Niwa et al. [75] failed to identify target mRNAs of Ire1 other than *HAC1*u mRNA. Accordingly, *IRE1*- and *HAC1*-gene knockout mutations result in the same phenotypes and do not exhibit additive or synergistic effects [76]. Whereas Tam et al. [77] reported that Ire1 cleaves a few types of mRNA other than *HAC1*u mRNA in *S. cerevisiae cells*, this observation has not been reproduced by others [78].

## 6. The UPR Signaling Pathway in Other Yeast and Fungal Species

Ire1 is conserved throughout eukaryotes and is likely to commonly act as an ER stress sensor in eukaryotic cells. One prominent downstream event of Ire1 activation is the splicing of mRNAs encoding metazoan XBP1, plant bZIP60, and fungal Hac1 [79,80]. The translation products of the spliced mRNAs are transcription factors responsible for transcription induction to cope with ER stress. Another role of Ire1 is to cleave certain mRNA species, which are then degraded without being rejoined. Whereas this phenomenon, namely the regulated Ire1-dependent decay (RIDD), was initially found in a Drosophila study, a wide variety of eukaryotic species are now known to perform it [80,81,82]. Ire1 is believed to preferentially cleave mRNAs encoding ER-client proteins, implying that a physiological role of RIDD is to decrease the protein load on the ER.

**Figure 6:**
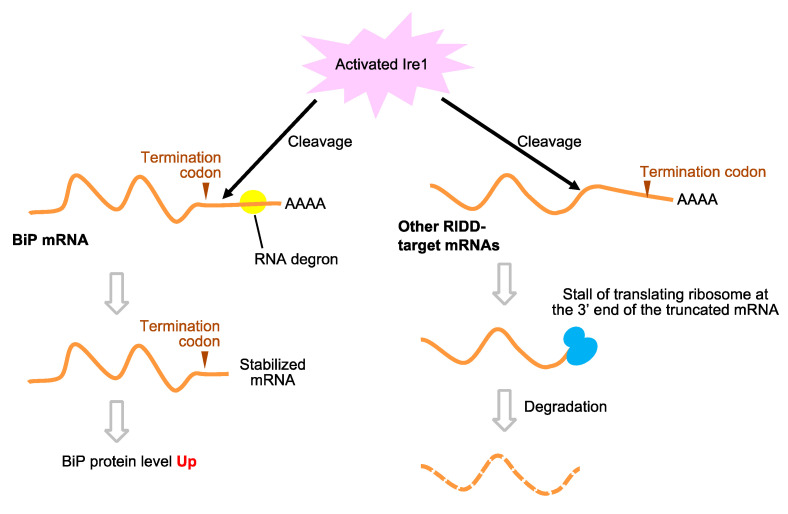
RIDD in *S. pombe* cells. In ER-stressed *S. pombe* cells, while Ire1 promotes the degradation of various target genes, BiP mRNA is stabilized via its cleavage by Ire1.

Unlike many other yeast and fungal species, *Schizosaccharomyces pombe* does not possess the *HAC1* ortholog. Kimmig et al. [83] proposed that the role of *S. pombe* Ire1 is to perform RIDD (Figure 6). The expression of a wide variety of mRNAs encoding ER client proteins is downregulated under ER stress conditions depending on Ire1 [83]. Guydosh et al. [84] proposed that after being cleaved by Ire1, mRNAs undergo no-go mRNA degradation (Figure 6). According to Li et al. [85] and Li et al. [86], the functional difference between *S. cerevisiae* Ire1 (the *HAC1* mRNA splicing) and *S. pombe* Ire1 (the RIDD) is due to the structural difference in their endoribonuclease domains.

Ironically and interestingly, unlike other Ire1-target mRNAs, BiP mRNA is induced dependently by Ire1 in ER-stressed *S. pombe* cells [83]. According to Kimmig et al. [83], this is because the 3′-UTR of *S. pombe* BiP has an unstabilizing sequence, which is removed by RIDD (Figure 6). Zhao et al. [87] demonstrated that in ER-stressed *S. pombe* cells, Ire1 also contributes to the induction of Epr1, which promotes ER-phagy.

Other yeast species, such as *P. pastoris*, *Hansenula polymorpha*, *Kluyveromyces lactis*, *Yarrowia lipolytica*, *Candida albicans*, and *Candida parapsilosis*, have been reported to carry *HAC1* orthologs, the transcripts of which are spliced in response to ER stress [88,89,90,91,92,93,94]. The intron sequence and length vary considerably between species. As well as the *HAC1* intron of *S. cerevisiae* (252 bp), those of *H. polymorpha* (177 bp) and *K. lactis* (297 bp) are likely to intramolecularly hybridize to the 5′-UTR, presumably leading to the translational attenuation of *HAC1u* mRNA versions [90,91]. In contrast, the lengths of the *HAC1* introns of *Y. lipolytica* and *C. albicans* are only 29 and 19 bp, respectively.

Some filamentous fungi belonging to *Aspergillus*, *Trichoderma*, or *Trichophyton* genera have also been reported to have *HAC1* orthologs, which are frequently called hacA based on their naming rules [95,96]. Saloheimo et al. [95] proposed that, in addition to the Ire1-dependent removal of the intron, *Aspergillus nidulans* hacA mRNA and *Trichoderma reesei* hac1 mRNA are truncated to remove their 5′-UTR, which acts to suppress their translation, under ER stress conditions. According to Mulder and Nikolaev [97], as a positive feedback loop, the HacA protein causes hacA gene transcription from a new start site proximal to the initiation ATG codon in *Aspergillus* cells. Tanaka et al. [98] and Yokota et al. [99] demonstrated that in *Aspergillus oryzae*, the UPR copes with ER stress induced by the secretion of hydrolytic enzymes.

The target genes of the Ire1-*HAC1* UPR signaling pathway are not the same among species. In *P. pastoris* cells, whereas the UPR highly induces genes encoding ER-located molecular chaperones and protein-folding enzymes, those encoding membrane lipid biogenesis do not appear to be prominent UPR targets [78,100]. Therefore, unlike in *S. cerevisiae* cells, the expansion of the ER membrane may not be an outcome of the UPR in *P. pastoris*.

Although they are not as strong as mutant proteins, normal secretory proteins induce ER stress and trigger UPR when highly expressed in a wide variety of eukaryotic cells. In agreement with the notion that *P. pastoris* has a robust and well-developed protein secretory system, the UPR is modestly, but clearly induced in non-stressed wild-type *P. pastoris* cells [89,101]. Consistently, the deletion of *IRE1* or *HAC1* retards the growth of *P. pastoris* cells even under non-stress conditions [78,89].

Presumably because of high-level protein secretion during an infection, the virulence traits of pathogenic yeasts and fungi are tightly linked to the UPR. The deletion of *IRE1* or *HAC1* considerably compromise the hyphal formation and virulence of *C. albicans* [93,102,103]. In addition, the hacA disruptant of *Aspergillus fumigatus* exhibits a reduction of protease secretion and virulence in mice [104]. Bitencourt [96] proposed the involvement of HacA in the virulence of *Trichophyton rubrum*. It should also be noted that UPR deficiency in pathogenic yeasts and fungi increases their susceptibility to azole antifungals, probably through the attenuation of ergosterol biosynthesis [96,102,105].

Therefore, the inhibition of the UPR may be a therapeutic strategy against pathogenic yeast or fungi [106]. For instance, a small organic compound, 4µ8C, which is known to selectively bind to and inhibit the major paralog of mammalian Ire1, IRE1α [107], also functions in *A. fumigatus* cells [108]. The UPR was blocked when *A. fumigatus* cells were incubated with 4µ8C [108]. *A. fumigatus* cells failed to grow on a collagen substrate in the presence of 4µ8C, implying a loss of virulence [108]. Moreover, 4µ8C increased the sensitivity of *A. fumigatus* to antifungal compounds [108]. Although it is uncertain whether 4µ8C is actually used as a therapeutic drug, these insights strongly suggest the potential of UPR-controlling chemicals against fungal infections.

*Cryptococcus* yeasts have another bZIP transcription factor, Hxl1, whose mRNA is spliced by Ire1, and then translated into an active transcription factor [109,110,111]. The Ire1/Hxl1-driven UPR is likely to contribute to the virulence of *Cryptococcus* species.

Nevertheless, studies on some yeast or fungal species have suggested that Ire1 also plays a role(s) other than the splicing of *HAC1* or *HXL1* mRNA [78,103,105,110]. Fauzee et al. [78] demonstrated that in *P. pastoris*, the *IRE1*-knockout and *HAC1*-knockout mutations result in only partially overlapping gene-expression alterations. *P. pastoris* Ire1 is likely to control the protein folding status in the cytosol independently of *HAC1* [78].

## 7. Engineering of the UPR for Application Purpose

As described thus far, the Ire1/*HAC1*-driven UPR transcriptionally induces the folding and modification of ER client proteins. Therefore, the artificial and constitutive induction of the UPR is anticipated to enhance ER function to mature secretory proteins.

Guerfal et al. [112] demonstrated that the productivity of secretory and cell surface proteins was increased by the artificial and high-level expression of the Hac1 protein in *P. pastoris*. As reviewed by Raschmanová et al. [113], several similar trials have been conducted to enhance the production of recombinant secretory proteins from *P. pastoris*. However, the overexpression of the Hac1 protein in *P. pastoris* does not always lead to favorable outcomes [112,113]. In some cases, the Hac1 orthologs of other yeast/fungal species or mammalian XBP1 protein were likely to be more effective than the authentic *P. pastoris* Hac1 protein was in improving the secretory protein productivity of *P. pastoris* [114]. Zahrl et al. [115] reported that the artificial expression of Hac1 protein highly enhanced secretory protein productivity when combined with the overexpression of Msn4, which is a transcription factor involved in another stress response pathway.

Although it occurs less frequently than *P. pastoris*, *S. cerevisiae* is often used for the production of recombinant secretory proteins. Probably because the UPR transcriptionally changes the expression of many genes, its abolishment has complicated effects on the production of human antibodies [116]. The intriguing biotechnological modification of *S. cerevisiae* cells is the heterologous production of polysaccharide-digestion enzymes, which may enable *S. cerevisiae* to use polysaccharides for ethanol fermentation without saccharification. According to Valkonen et al. [117] and Bao et al. [118], the secretion of heterologous α-amylase and xylanase was moderately enhanced by the artificial expression of Hac1 protein.

Nevertheless, as described thus far, an unregulated UPR is harmful, at least in *S. cerevisiae*. Therefore, the artificial and constitutive expression of Hac1 protein severely retards the growth of *S. cerevisiae* [119,120]. We speculate that in some reports, Hac1 protein was expressed only moderately, leading to a modest UPR that retarded cellular growth and increased protein secretion only slightly.

How can we benefit more from artificial UPR induction without its harmful effects? Lin et al. [121] reported the improvement of secretory protein production via the overexpression of factors that assist the Hac1 protein production, but not via the overexpression of Hac1 protein per se. On the other hand, Nguyen et al. [120] reported that the growth of *S. cerevisiae* cells artificially producing the Hac1 protein was accelerated by their exposure to weak ER stress. This is presumably because weak ER stress moderately yields unfolded proteins, which capture excessively expressed molecular chaperones in the ER.

**Figure 7:**
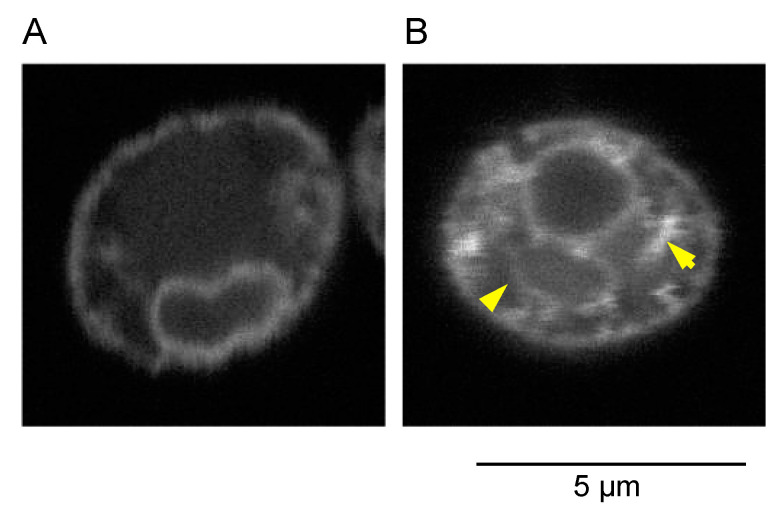
ER expansion in *S. cerevisiae* cells artificially expressing *HAC1*i mRNA. As described in Nguyen et al. [121], *S. cerevisiae* BY4742 cells (**A**) or their variant expressing *HAC1*i mRNA (**B**) were transformed with an ER-located GFP-expression plasmid and observed under a confocal fluorescence microscope. The expanded ER is indicated by yellow arrowheads.

In addition to secretory proteins, lipid molecules are also prominent products of the ER. Nguyen et al. [120] demonstrated the drastic expansion of the ER throughout the cytoplasm in *S. cerevisiae* cells artificially expressing the Hac1 protein (Figure 7). Consistently, the production level of triglycerides and terpenoids was reported to be increased via the artificial expression of the Hac1 protein [120,122].

## 8. Future Perspective

Whereas the mainstream of the yeast UPR, which is governed by Ire1 and *HAC1*, seems to be well elucidated, some intriguing topics remain unanswered. According to Ho et al. [50] and Thibault et al. [123], the target genes of the UPR differ depending on the primary cause of stress. For instance, *ERO1* is strongly and transiently induced by DTT, but only modestly induced by LBS in *S. cerevisiae* [50]. We assume that the function of Hac1 can be modified by other factors, which should be investigated in the future. There is also an intriguing question concerning how UPR is involved in cytoprotection under various conditions such as high and low temperatures, DNA damage, and drought.

Another research question concerns a proximate target(s) of Ire1 other than *HAC1* mRNA. As mentioned above, *HAC1* mRNA splicing is likely to be the sole role of *S. cerevisiae* Ire1, whereas Ire1 of some other yeast and fungal species has been shown to also have another role(s). Although it is not evident, it may be RIDD; this is similar to the case of *S. pombe*. Alternatively, the kinase domain of Ire1 may function to phosphorylate other proteins in addition to the auto-phosphorylation of Ire1.

## Data Availability

Not applicable.
